# Characterization of intestinal microbiota and serum metabolites in patients with mild hepatic encephalopathy

**DOI:** 10.1515/biol-2021-0140

**Published:** 2022-03-10

**Authors:** Yong Lin, Gengjie Yan, Feng Feng, Minggang Wang, Fuli Long

**Affiliations:** Department of Liver Disease, The First Affiliated Hospital of Guangxi University of Traditional Chinese Medicine, Nanning, Guangxi 530023, China

**Keywords:** micro-hepatic encephalopathy, intestinal microbiota, serum metabolites, variable characteristics, interactive relationship

## Abstract

Mild micro-hepatic encephalopathy (MHE) is a severe complication of cirrhosis. At present, there are differences in the consistency of detection strategies and treatment directions for MHE. The characteristic changes in intestinal microbiota and serum metabolites in MHE patients and the possible relevant interaction mechanisms would inevitably affect the developmental direction of MHE. Therefore, the changes in the characteristics of intestinal microbiota and serum metabolites of MHE patients were determined, and the possible interactions between them were analyzed. Stool and serum tests were performed on both the MHE patients and healthy individuals. The 16S rRNA gene high-throughput sequencing and bioinformatics analyses were used to analyze the differences in intestinal microbiota in MHE patients. The serum metabolites were detected using liquid LC-MS/MS (liquid chromatography-mass spectrometry) technology, and the differences in the metabolic networks of blood metabolites in MHE patients were analyzed. A comprehensive bioinformatics analysis approach was adopted to identify the composition and characteristics of microbiota and serum metabolites and the possible correlation between them. The main characteristics of the structural imbalance in the intestinal microbiota of MHE patients included a decrease in the number of beneficial bacteria at the levels of phylum, class, order, family, and genus and an increase in the pathogenic bacteria, resulting in substantial changes in the relative abundances of bacteria in the intestinal microbiota. The main predicted functions that showed significant differences included chromosome, amino acid-related enzymes, methane metabolism, and arginine and proline metabolism. The detection of serum metabolites resulted in 10 different metabolites, including taurocholic acid, citrulline, d-phenyl-lactic acid, l-tyrosine, benzoate, phenylalanine, linoleic acid, eicosapedienic acid, alpha-dimorphecolic acid, and dehydroepiandrosterone. The subsequent metabolite pathways analysis showed differences in the metabolism of linoleic acid, phenyl-propane, caffeine, arginine, proline, glycine, serine, threonine, tyrosine, and pyrimidine compared to the control group. In summary, it seems that the changes in the microbiome that we have identified have resulted in corresponding changes to the serum metabolome. In turn, this may represent changes in the absorption of metabolites from the gut or reflect the changed metabolic capacity of the MHE liver or both. There were characteristic changes in the intestinal microbiota and serum metabolites in the MHE patients. There might be a related interaction mechanism between the two, which would provide evidence and direction for the detection and treatment strategies of MHE.

## 1 Introduction

Micro-hepatic encephalopathy (MHE) is a particular type of hepatic encephalopathy (HE), with usually no apparent signs and symptoms. Its mechanism of pathogenesis is very similar to that of HE but differs in the progression and severity of the disease [1,2]. The ammonia poisoning theory is the earliest hypothesis with the most evidence. Ammonia poisoning, caused by the disordered metabolism of ammonia, can trigger the cascade reaction of neuron apoptosis [3]. Both the MHE and dominant HE have different degrees of cognitive dysfunction but their pathologic basis is quite different, resulting in different diseases. MHE is mainly followed by cirrhosis, while the dominant HE is mostly followed by liver failure. It can be speculated that there might be certain differences in the pathogenesis of HE. Recent studies have found that cirrhosis is associated with changes in the composition and function of intestinal microbiota, which might accelerate the progression of multiple complications, including infections, such as MHE, spontaneous bacterial peritonitis (SBP), and renal dysfunction [4,5]. In addition, there are complex interactions between intestinal microbiota and the development of the brain and neurophysiological functions [6]. Literature evidence shows that the intestinal microbiota can directly affect the blood–brain barrier, myelin sheath, neurogenesis, maturation of microglial cells, and other basic neural development processes, regulating a variety of neurophysiological activities [7]. It also forms tryptamine, an arylamine neurotransmitter, which affects the acquisition of amino acids used in the synthesis of neuroactive peptides. It also regulates the exchange of peptide signals between the peripheral nervous system and brain through the blood–brain barrier and participates in the brain’s response to the signals generated by intestinal microbiota [8,9]. Effective intervention and control of imbalances in intestinal microbiota help delay and prevent the occurrence and development of liver diseases [10]. Similarly, the serum metabolites and related inflammatory factors are also reported to affect the occurrence and development of MHE and might have an interactive correlation with intestinal microbiota [11,12]. Studies on MHE cognitive impairment point toward the changes in the enterohepatic and cerebral axis, including changes in the intestinal microbiota, composition of serum metabolites, impaired immune response, and an increase in the severity of local and systemic inflammation [13,14]. Therefore, starting from intestinal microbiota and serum metabolites, this study investigated the correlation between the structure, function, and metabolism of different bacteria with MHE and the characteristic serum metabolites and metabolic pathways.

## 2 Methods

### 2.1 Research objects and criteria

In this population-based study, the stool and blood samples were collected from the MHE patients to characterize intestinal microbiota and serum metabolites, respectively. After obtaining informed consent, 16 MHE outpatients and 10 social volunteers were recruited from the liver disease clinic of the First Affiliated Hospital of Guangxi University of Traditional Chinese Medicine. The inclusion criteria consisted of the following: (1) The patients were diagnosed with post-hepatitis B cirrhosis using any of the following diagnostic methods: liver biopsy, transient elastography, evidence of varicose veins, and hepatic morphology or thrombocytopenia in patients with chronic liver disease or decompensated cirrhosis. (2) Diagnostic criteria for MHE patients conformed to the diagnostic criteria for post-hepatitis B cirrhosis. The Child-Pugh score for cirrhosis was classified into Classes A and B, where both the number connection test (NCT) and symbol digit test (SDT) or one of them were abnormal. (3) Healthy individuals (control group) were included in the study after a complete medical history, physical examination, chest X-ray, routine hematuria, blood glucose, liver, and renal function, and other physical and chemical examinations to determine whether they had any diseases of the heart, brain, liver, kidneys, and lungs or other major organ systems. (4) The patients included in this study were 18–65 years old. The patient signed the informed consent form. These individuals had no previous tips (transjugular intrahepatic portosystemic shunt). Patients with a history of liver cirrhosis, including patients with an unclear history of liver cirrhosis, current alcoholism or drug abuse, and patients taking antipsychotics, painkillers, antidepressants, or benzodiazepines, were excluded. Recent (within the last 3 months) patients with transjugular intrahepatic portal vein shunt, recent (within the last 3 months) patients treated with opioids due to changes, and recent (within 1 month) hospitalized patients were excluded.


**Informed consent:** Informed consent has been obtained from all individuals included in this study.
**Ethical approval:** The research related to human use has been complied with all relevant national regulations, institutional policies and in accordance with the tenets of the Helsinki Declaration, and has been approved by the Ethics Committee of the First Affiliated Hospital of Guangxi University of Traditional Chinese Medicine (Ethics No. 2018-002-01).

### 2.2 Research methods

The diet plan of the 26 included subjects for the first three days of specimen collection was uniformly managed by emphasizing the intake of calories, proteins, meat, and vegetables. Fresh stool (200–300 mg) and 5 mL of fasting venous blood were collected from the subjects in the morning. Invitrogen DNA Mini Kit was used to extract DNA from stool samples, quantified using a fluorescence meter, while its integrity was tested using the E-Gel electrophoresis system. The composition of intestinal microbiota was analyzed using 16S rRNA gene sequencing in two steps, according to Gillevet and Hamady [15,16]. The Kyoto Encyclopedia of Genes and Genomes (KEGG) metabolic pathways spectrum analysis was used to select OTU sequence at 97% similarity level using QIIME software. The output script was analyzed using PICRUSt software to analyze the frequency of metabolic functions at KEGG pathway levels. The analysis of serum metabolomics was performed using the published technique, liquid chromatography-mass spectrometry (LC-MS/MS) [17].

### 2.3 16S rRNA analysis

#### 2.3.1 16S rRNA high-throughput sequencing

According to the operation instructions of the fecal genomic DNA extraction kit, the total microbial DNA in fecal samples of mice in each group was extracted. The integrity of DNA was detected by agarose gel electrophoresis. The V3–V4 region of bacterial 16S rRNA gene was amplified by PCR with the target sequence reflecting the composition and diversity of flora as the target, and the forward primer (5′-aytggydtaaagng-3′) and reverse primer (5′-tacnvggtatctaatc-3′) with bar code specificity were added as amplification primers, The amplified products were recovered by 2% agarose gel electrophoresis and purification of magnetic beads. The library was built with truseq nano DNA LT library prep kit of Illumina Company, and the library was sequenced with high-throughput on miseq platform.

#### 2.3.2 Bioinformatics analysis

Flash 1.2.7 software was used to screen and control the quality of the original sequencing data, merge, and calculate the classification unit (OTU) according to 97% sequence similarity, and analyzed the alpha diversity of the sample community by analyzing the Chao1 index reflecting the community richness and the Shannon diversity index reflecting the community evenness; Qiime 1.8.0 software was used to obtain the composition and abundance distribution table of each sample at the phylum and genus classification levels; mothur 1.18.0 software and metastats statistical algorithm were used to compare the abundance differences of each taxon at the phylum and genus levels between groups. Beta diversity analysis projects the original high-dimensional data (such as flora OTU abundance matrix) to the spatial coordinate system with lower dimension through linear transformation and combination and through R 3.6.1 software. Principal component analysis (PCA) was used to analyze the community composition structure at the genus level, and two-dimensional images were used to describe the natural distribution characteristics among samples, so as to quantify the differences and similarities between samples. According to the known microbial genome data, the sequencing data of flora composition (the sequencing results of 16S rRNA gene) were used to predict the metabolic function of flora.

### 2.4 LC-MS/MS analysis

The reasons for the inconsistency between 16SrRNA and LC-MS/MS samples are as follows. Because our experimental arrangement is to detect 16SrRNA first (10 cases in the normal group and 16 cases in the treatment group), and then detect LC-MS/MS. During the preservation of patient samples, there are some factors that may contaminate the samples. Considering the possible errors, there were only 9 normal groups and 12 treatment groups in LC-MS/MS test.

#### 2.4.1 Sample pretreatment

The sample was thawed at room temperature and sucked for 100 min with a pipette gun μ. One serum sample was put into 1.5 mL EP tube. About 300 μL of methanol and 10 μL of internal standard (2.5 mg/mL, 2-chlorophenylalanine) were added. The solution was vortex mixed for 30 s, placed in a 4°C centrifuge, and centrifuged for 15 min at 12,000 rpm. The absorbed 200 μL supernatant was transferred into the injection vial for detection.

#### 2.4.2 Test conditions

Waters Acquity UPLC HSS T3 column (2.1 mm) was used (100 mm × 1.8 μm). The mobile phase was 0.1% formic acid in aqueous solution (a) acetonitrile (b) gradient elution (0–2 min, 95% a, 5% B; 2–12 min, 95– 5% a, 5–95% B; 12–15 min, 5% a, 95% B; 15–17 min, 5–95% a, 95–5% B; 17–20 min, 95% a, 5% B). The flow rate was 0.3 mL/min and the injection volume was 6 μL. The column temperature was 40°C. ESI + and ESI scanning mass spectrometry were used; the mass scanning range was 50–1,500 m/z, and the capillary voltages were 1.4 and 1.3 kV. The taper hole voltages were 40 and 23 V; the ion source temperature was 120°C; the desolvent gas temperature was 350°C; the conical hole gas flow was 50 L/h; the desolvent gas flow was 600 L/h; the collision energy range was 10–40 V; and the ion energy was 1 V. The spectrum was collect every 0.2 s. Rutin solution was used as the locking mass solution for accurate mass determination.

#### 2.4.3 Data analysis and metabolite identification

First, the original data were transformed into CDF format file by cdfbridge in masslynx 4.1 workstation software, and then the xcms package was used for peak extraction, peak comparison, peak filtering, supplement of missing peaks and other data processing. The data were finally standardized into a two-dimensional data matrix in Excel format. Before formal analysis by smica-p software, the data group was normalized for more intuitive and reliable results. The differential variables were screened by PCA and PLS-DA, combined with s-plot, PLS-DA model variables, variable importance projection value (VIP), and *t*-test.

### 2.5 Statistical analysis

All of the data were expressed as mean ± standard deviation. Levene’s test, analysis of variance (ANOVA), and Students–News–Keuls test were used for the homogeneity of variance test, comparison between groups, and pair-wise comparisons between groups, respectively (Kruskal–Wallis H test was used for the comparison between groups with uneven variances, and Wilcoxon Rank Sum test was used for the pair-wise comparison between groups). The multifactor non-bar logistic regression analysis and backward stepwise selection were adopted for the simultaneous calculation of the odds ratio (OR) and 95% confidence interval (95% CI) of all the factors. The Spearman’s rank correlation coefficient was used for the correlation analysis of the normal distribution of data (SPSS 22.0 was used for the correlation analysis of non-normal distribution data). *p* < 0.05 was considered statistically significant.

## 3 Results

### 3.1 Analysis of the intestinal microbiota using 16S rRNA gene sequencing

#### 3.1.1 Alpha and beta diversity analyses of the intestinal microbiota differences in MHE patients

**Figure 1:**
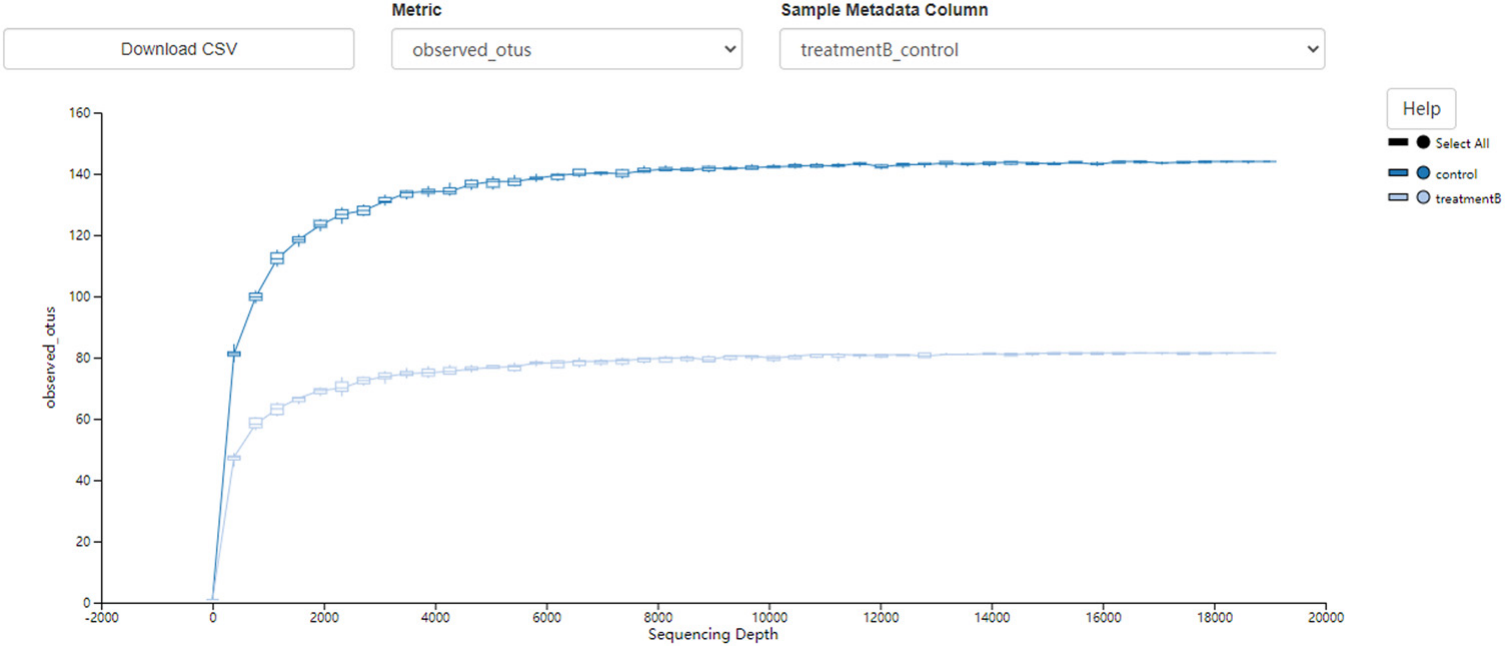
The sequence depth vs the abundance of species.

**Figure 2:**
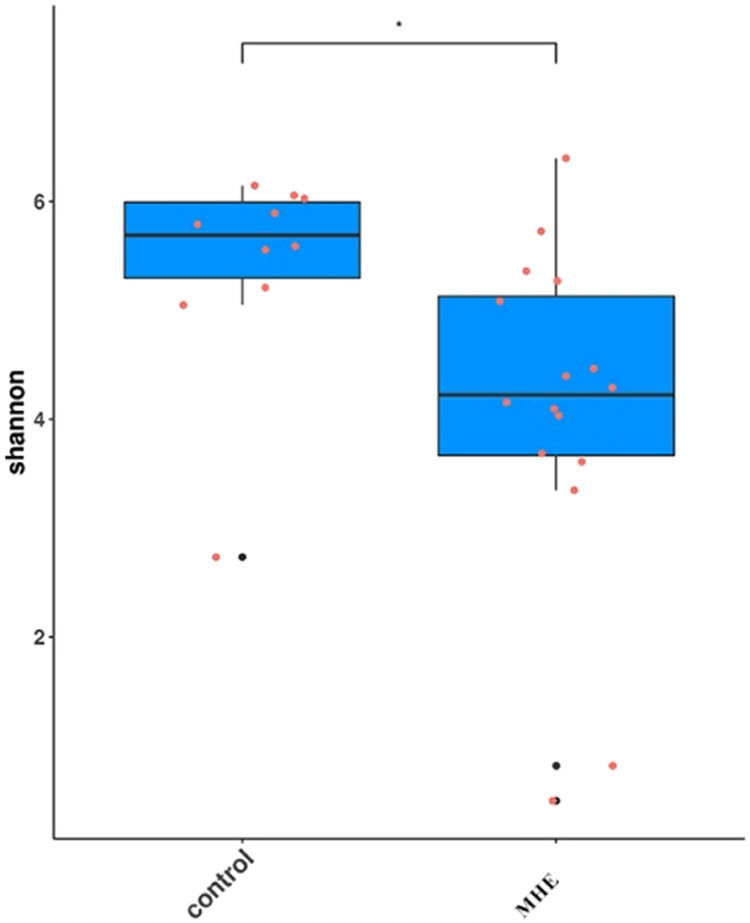
Shannon community diversity index between groups. Note: The *X*-axis represents the group name and *Y*-axis represents the alpha diversity index’ *p < 0.05.

**Figure 3:**
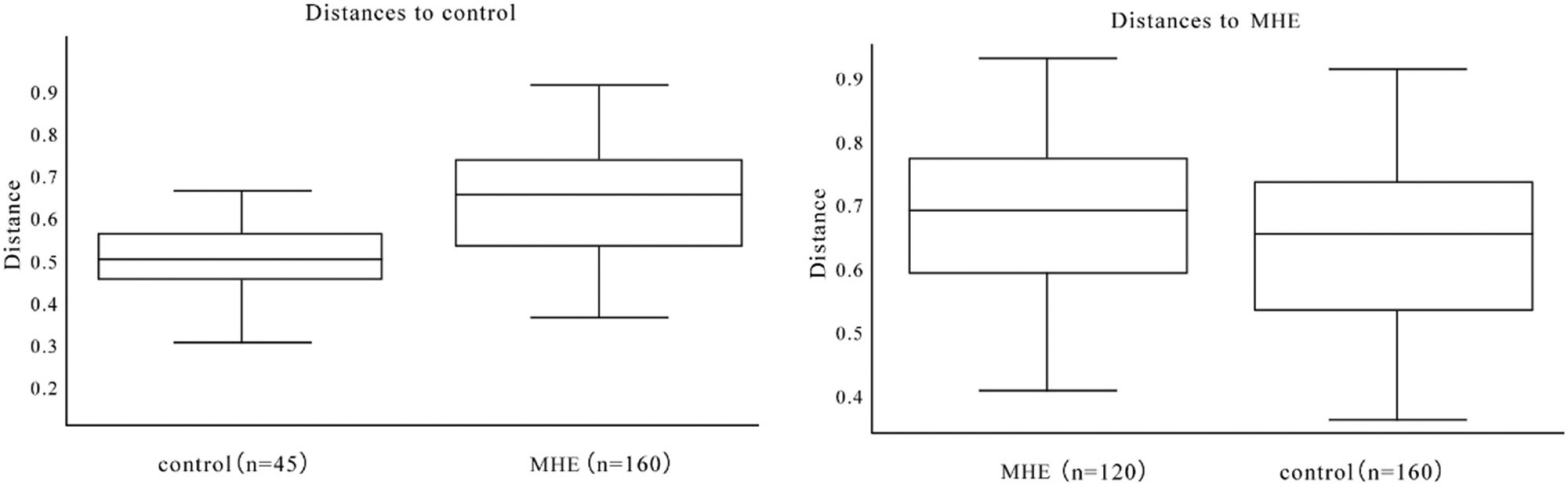
The box diagram shows the grouping information horizontally, and the *n* values in parentheses after each grouping name represents the comparison times between the two groups; the vertical line is the average of the two groups.

The alpha and beta diversity analyses were used to analyze the significance of differences in microbial composition between the two sample groups. The alpha index was used to analyze the richness and diversity of microbial communities. As shown in Figures 1 and 2, the alpha curve flattened with an increase in sample size, indicating that the sample size was sufficient to reflect the richness of the species community and other changes. The sequencing depth required for the curve of the control group to reflect the richness of the species community was higher than that of the MHE group, indicating lower species richness in the MHE group. Similarly, in Table 1, both the alpha diversities, including Chao1 and Shannon indices, were reduced (*p* < 0.01), indicating that the richness and diversity of intestinal microbiota in the MHE group decreased significantly with significant differences in the structure of an individual’s intestinal microbiota. The subsequent box diagram (Figure 3) and corrected *p* values (Table 2) also confirm these conclusions.

**Table 1:** Alpha index of diversification ( \bar{x}\pm S] )

Group	Chao1	Shannon
Control	149.00 ± 44.32	5.41 ± 1.01
Treatment B	84.53 ± 4.01**	3.80 ± 1.52**

Note: **p <0.01 was compared with the control group.

**Table 2:** PERMANOVA results of two groups

Group1	Group2	Sample size	Permutations	*p*-value	*p*-value
Control	Treatment B	26	999	0.002	0.002

#### 3.1.2 Analysis of the differences in the taxonomic composition of intestinal microbiota in the MHE patients at the levels of phylum, class, order, family, and genus

**Figure 4:**
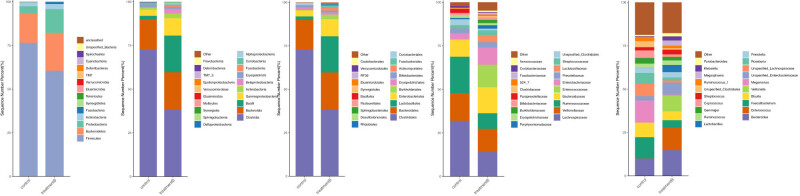
The taxonomic composition and abundance distribution of the communities at the levels of phylum, class, order, family, and genus.

According to the results of species (as shown in Figure 4), at the levels of phylum, class, order, family, and genus, *Firmicutes* and *Proteobacteria*; *Clostridia*, *Coriobacteriia*, and *Bacilli*; *Lactobacillales*, *Clostridiales*, *Actinomycetales*, and *Coriobacteriales*; *Lachnospiraceae* and *Coriobacteriaceae; Roseburia, Lachnospiraceae, Coprococcus*, and *Veillonella*, respectively, showed statistically significant differences in their relative frequencies between the two groups (Table 3, *p* < 0.05). To further explore the intestinal microbiota in MHE patients at the genus level, the top 20 OTUs were ranked in horizontal frequency, which showed the frequencies of *Bacteroides, Enterococcus, Faecalibacterium, Blautia, Veillonella, Megamonas, Unspecified_Enterobacteriaceae, Unspecified_Lachnospiraceae, Roseburia, Prevotella, Lactobacillus, Ruminococcus, Gemmiger, Coprococcus, Streptococcus, Unspecified_Clostridiales, Ruminococcus, Megasphaera, Klebsiella,* and *Parabacteroides*.

**Table 3:** Comparison of the MHE group and control group at the levels of phylum, class, order, family and genus

Phylum	Class	Order	Family	Genus	*p*-value	Control ( \bar{x}] )	Treatment *B*( \bar{x}] )
Fimicutes					0.026	0.765	0.605
Proteobacteria					0.039	0.040	0.137
	Clostridia				0.000	0.729	0.384
	Bacilli				0.03	0.021	0.211
	Coriobacteria				0.036	0.001	0.003
		Clostridiales			0.000	0.729	0.384
		Lactobacillalcs			0.03	0.021	0.211
		Coriobacteriales			0.036	0.001	0.003
		Actinomycctalcs			0.048	0.000	0.001
			Coriobacteriaceae		0.036	0.001	0.003
			Lachnospiraceae		0.009	0.321	0.140
				*Veillonella*	0.022	0.000	0.091
				*Lachnospiraceae*	0.017	0.072	0.012
				*Roseburia*	0.026	0.062	0.017
				*Coprpcpccus*	0.026	0.043	0.014

#### 3.1.3 Correlation statistical analysis of the differences in intestinal microbiota

**Figure 5:**
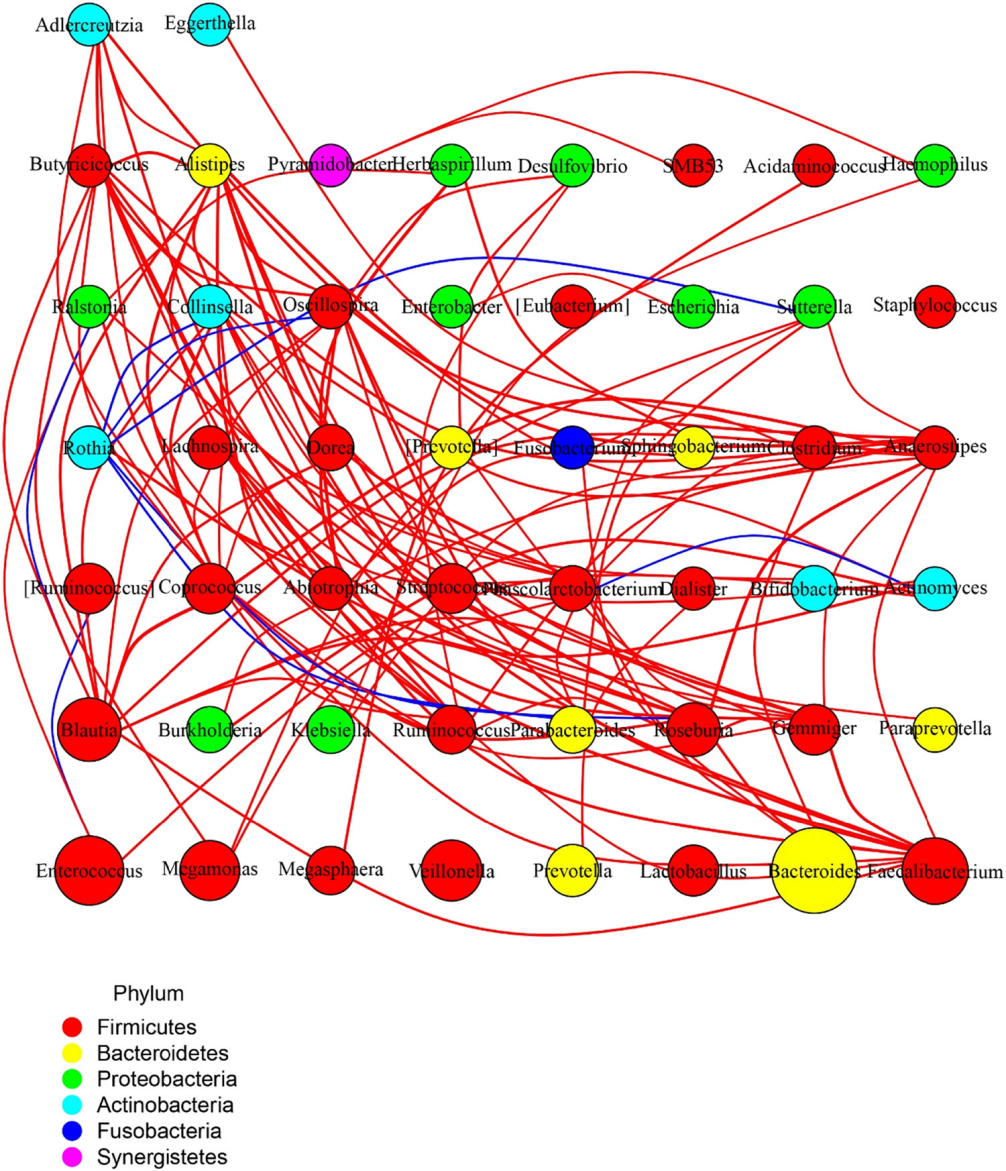
Spearman’s rank analysis to construct the antagonistic or cooperative species information through the network. Each circle represents a species, the size represents the abundance, the lines in red are positively correlated, the lines in blue are negatively correlated. and the thickness represents the correlation coefficient.

The differences in the composition of intestinal microbiota between the MHE and healthy group were analyzed using the above-mentioned analysis; however, the correlation of the interaction between the microbiota in the same individual was still unclear. Therefore, Spearman’s rank analysis was conducted based on the relative frequencies of the genus in the same sample to construct the antagonistic or cooperative species information through the network (Figure 5). It was found that *Rothia* was significantly and negatively correlated with *Oscillospira, Collinsella, Parabacteroides,* and *Roseburia*. Similarly, *Enterococcus, Ruminococcus,* and *Ralstonia* exhibited a significant negative correlation with *Blautia*, *Phascolarctobacterium,* and *Actinomyces*.

#### 3.1.4 Analysis of the function of microbial communities of different intestinal microbiota

**Figure 6:**
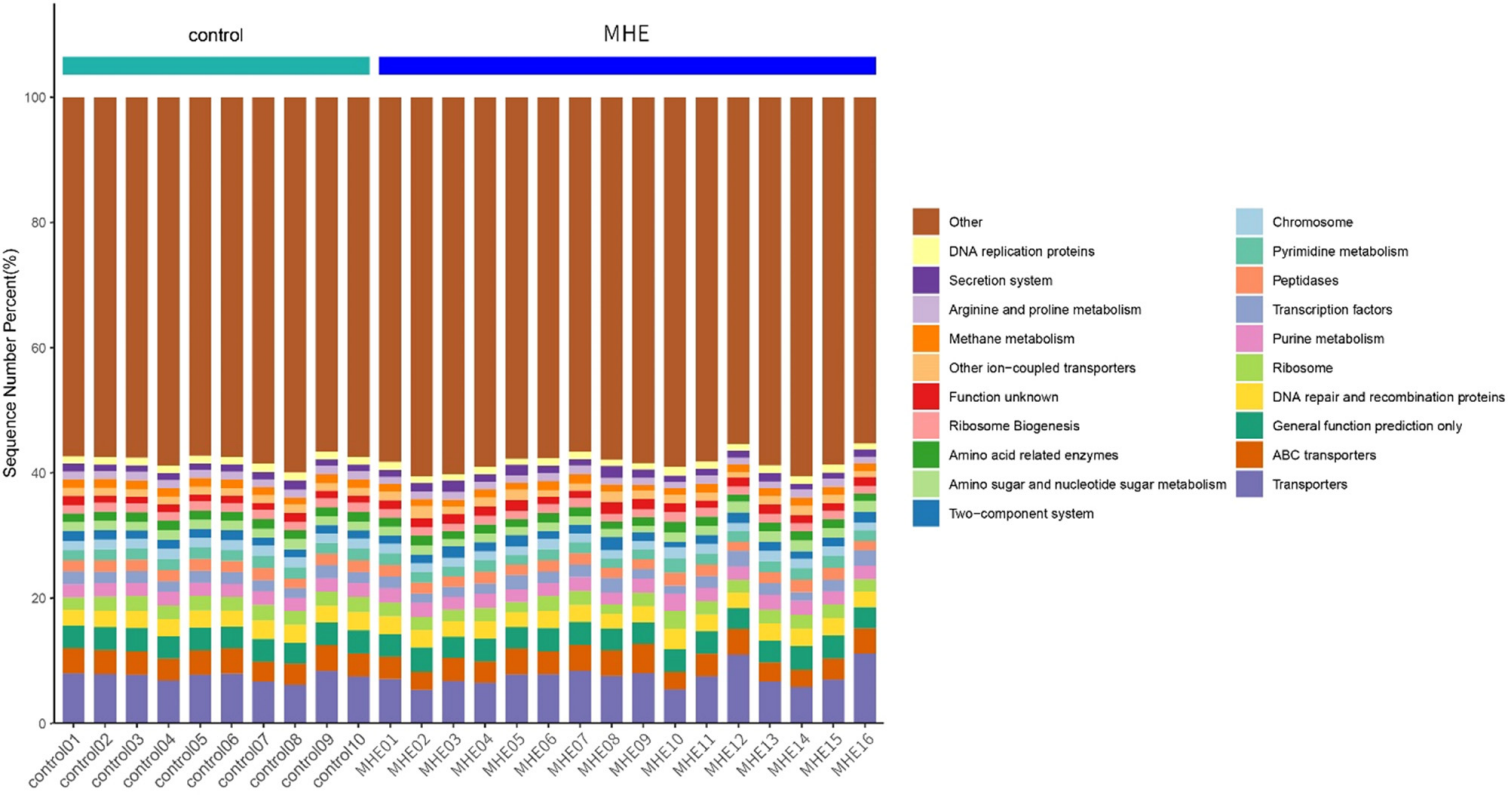
Bar chart composed of KEGG L3 level functional prediction.

Figure 6 shows the Greengenes database-based PICRUSt analysis in which the spectrum of gene functions in the species was inferred, and then the functions of the whole spectrum genes were predicted. Finally, the composition of intestinal microbiota was mapped to the KEGG database L3 bar charts. Table 4 shows the statistical data at the level of L3 function prediction. It can be seen that there were significant differences in the predicted function between MHE and control groups, including the differences in chromosomes, amino acids, methane metabolism-related enzymes, arginine, and proline metabolism (*p* < 0.05).

**Table 4:** Function prediction of KEGG L3 microbial community ( \bar{x}\pm S] )

Group	Chromosome	Amino acid related enzymes	Methane metabolism	Arginine and proline metabolism
Control	0.0157 ± 0.0006	0.0148 ± 0.0006	0.0134 ± 0.0011	0.0129 ± 0.0004
Treatment B	0.0148 ± 0.0013*	0.0139 ± 0.0016*	0.0121 ± 0.0007*	0.0119 ± 0.0012**

Note: *p < 0.05, **p < 0.01, compared with the control group.

### 3.2 Analysis of serum metabolites based on LC-MS/MS

#### 3.2.1 Data preprocessing

**Figure 7:**
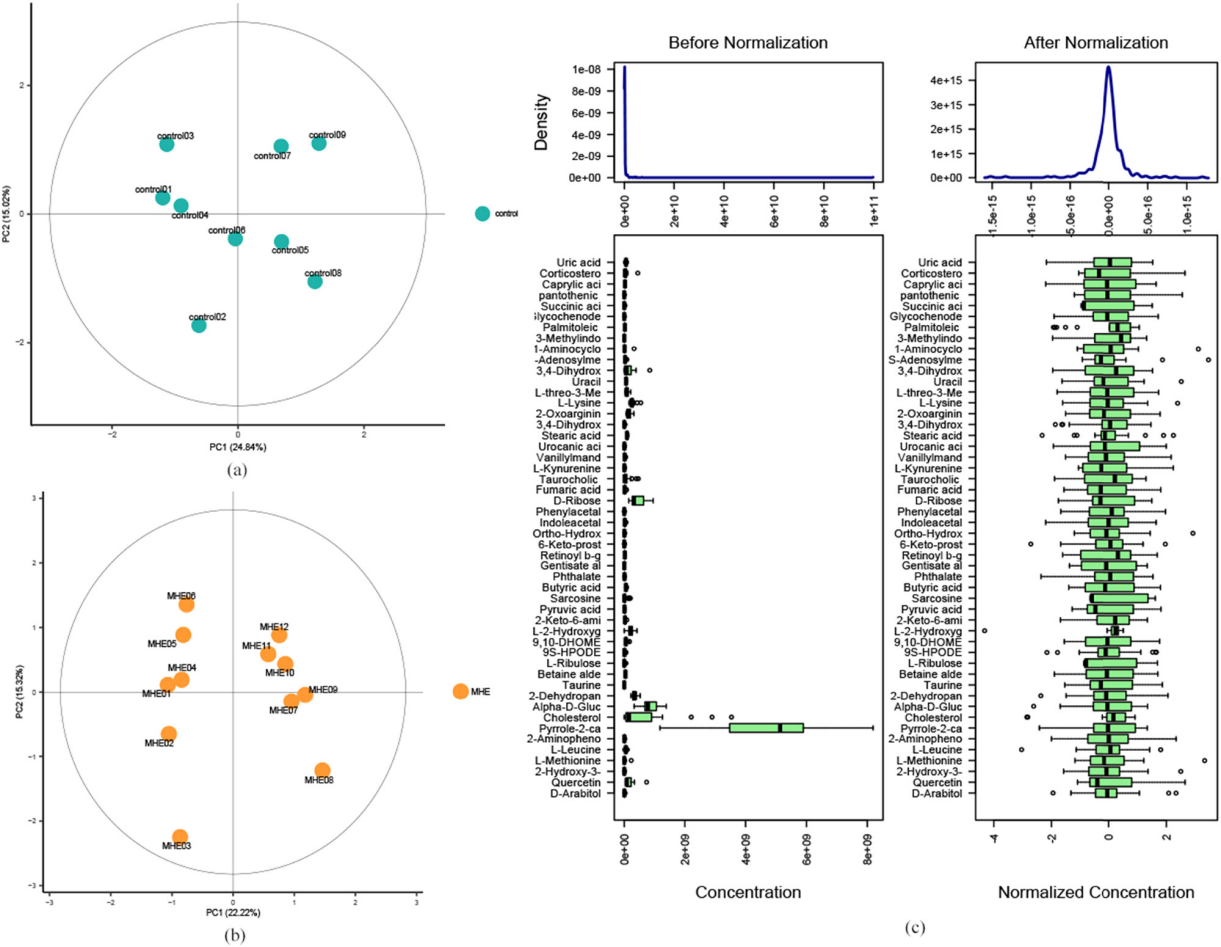
PCA scores of the (a) the control and (b) the MHE. (c) The content distribution of each metabolite before and after standardization.

The quality assessment (QA), quality control (QC), and standardized processing were conducted on the sample data (Figure 7(a) and (b)) to ensure the reliability of data and reduce the error in the measurement system. Figure 7(c) shows that the principal components analysis (PCA) scores of both the groups were within 95% CI, which validated the standardization. Figure 7 shows that the distribution of the metabolite contents of the samples after standardization was close to the normal distribution, which was suitable for the subsequent PCA, partial least-squares discrimination analysis (PLS-DA), orthogonal PLS-DA (OPLS-DA) analyses, *t*-test, and ANOVA tests.

#### 3.2.2 Differential analysis between MHE and control groups

**Figure 8:**
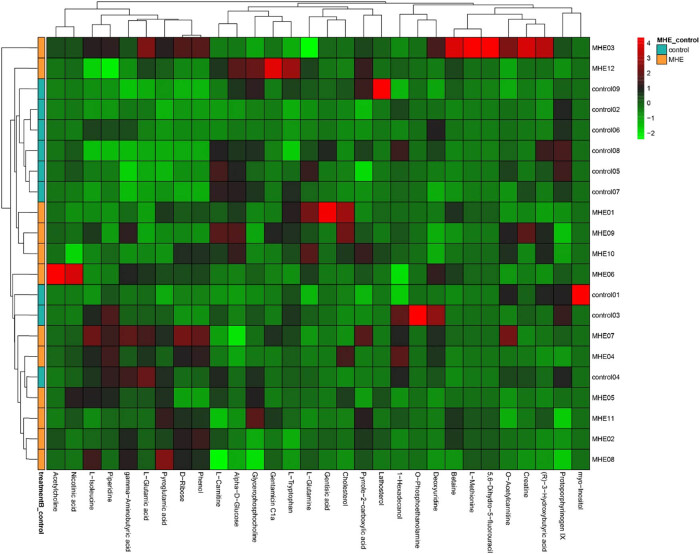
Results of the metabolite heat map clustering.

**Figure 9:**
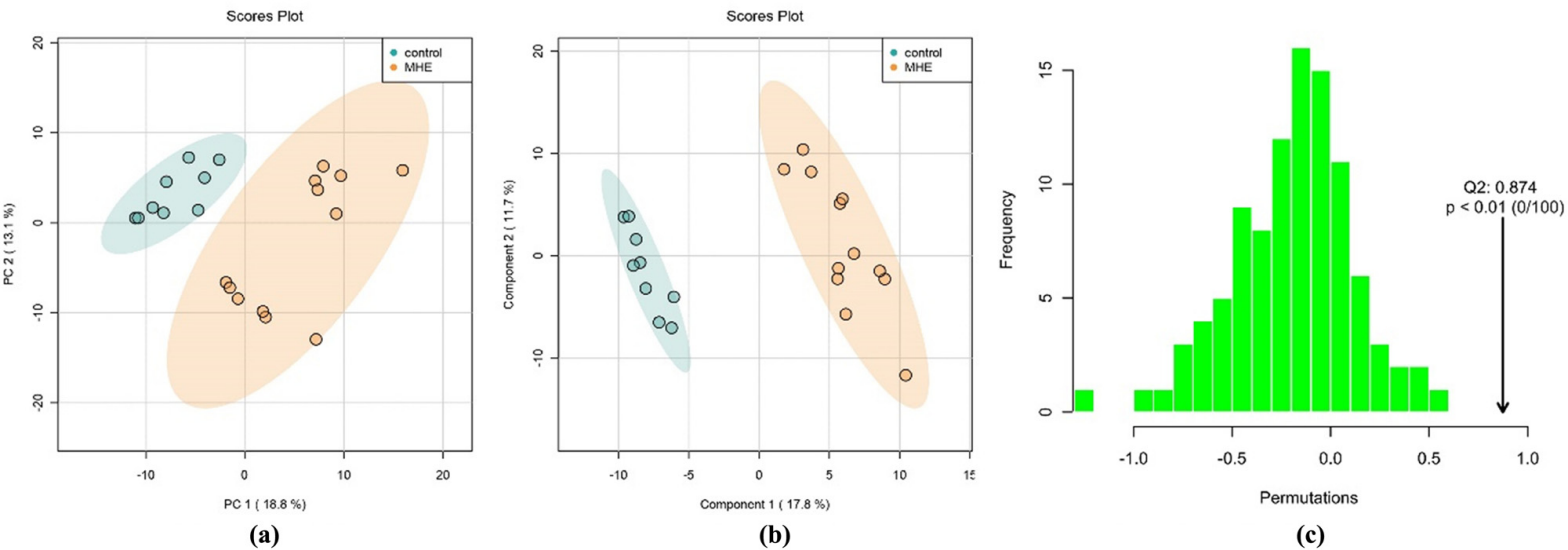
*p* values of (a) PCA, (b) PLSDA point cloud graph, and (c) Q2 distribution of OPLS-DA test statistics.

Significant differences can be seen from the heat-maps of the clustering of metabolites between the MHE and control groups (Figure 8), among which the clustering of metabolites, such as pyroglutamic acid, d-ribose, phenol, and gentamicin C1a in the MHE group was more obvious than that in the control group. The thermal metabolites, including protoporphyrinogen IX, O-phosphoethanolamine, Myo-inositol, l-carnitine, etc., were more concentrated in the control group. As shown in the heat-maps of the clustering results, only the rough differences in the metabolites between groups, the subsequent accurate PCA, PLSDA, and OPLSDA analysis were conducted. The PCA and PLSDA point cloud map showed significant separation between the two groups and significant differences in metabolites between the two groups (Figure 9(a) and (b)). In Figure 9(c), OPLS-DA permutation test Q2 was 0.87, and the actual observed Q2 indicated by the arrow is on the right side of the random distribution. The predictive ability of the model was significant, indicating significant differences in metabolites between the two groups.

#### 3.2.3 Screening and identification of intergroup characteristic metabolites

**Figure 10:**
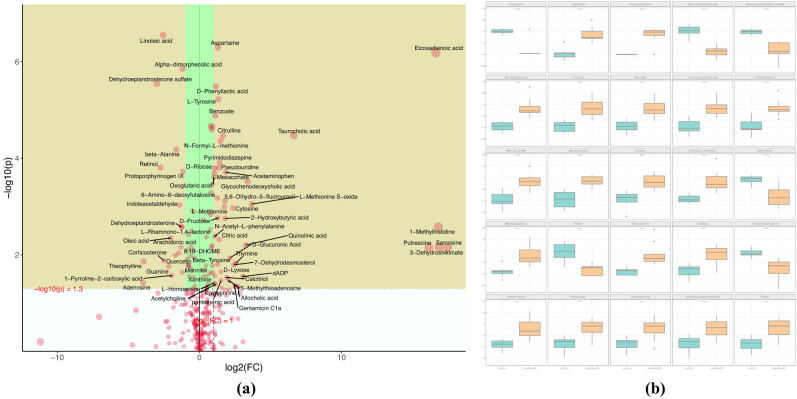
(a) Volcanic diagram with multiple changes and (b) block diagram of the first 25 metabolite differences (* *p* < 0.05, ** *p* < 0.01, *** *p* < 0.001).

**Figure 11:**
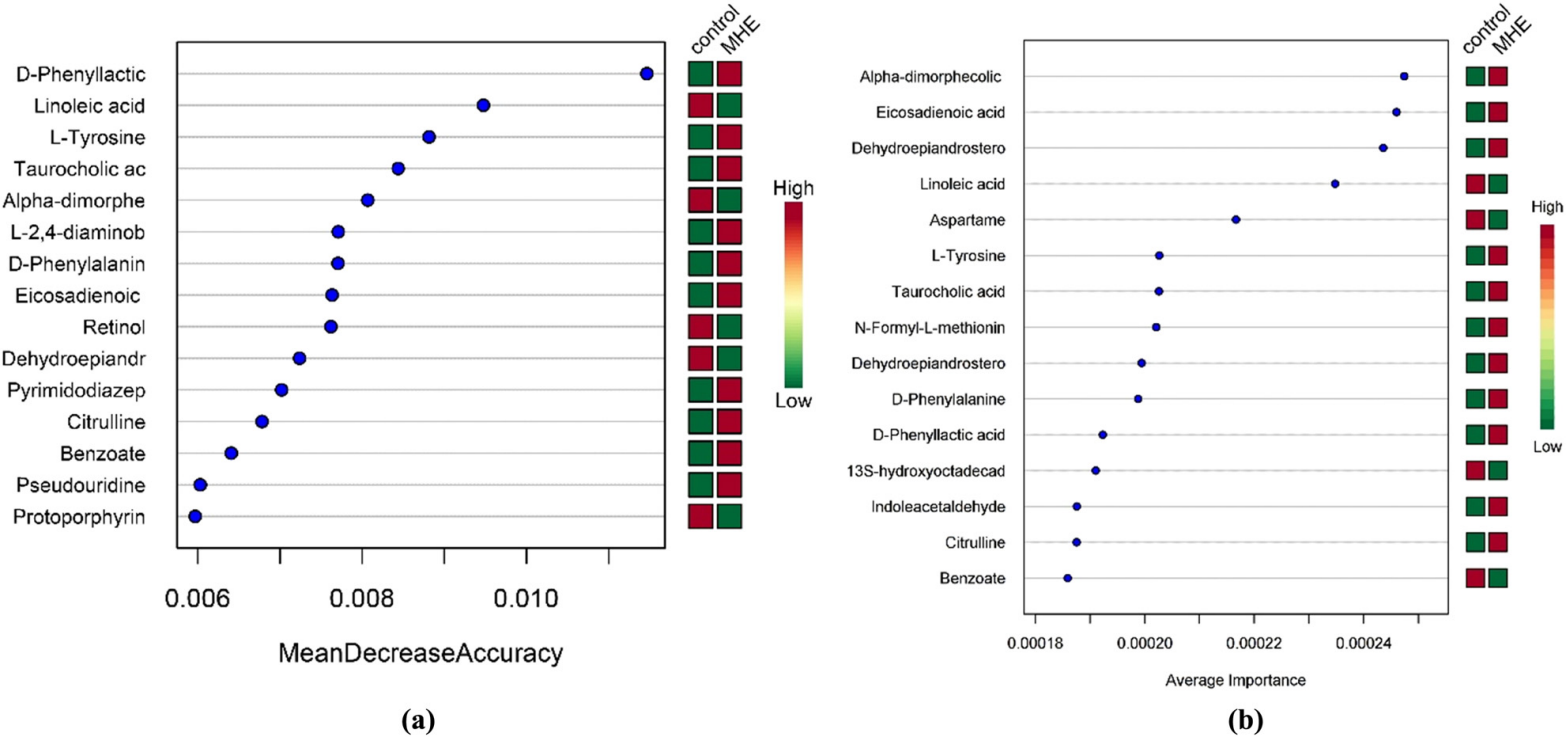
(a) Fifteen metabolites in the random forest and (b) 15 metabolites in the SVM.

Three different ways were selected to compare the characteristic metabolites between groups to reduce the influence of different experimental methods and increase the validity of the results, and the same differential metabolites were screened out in two groups. In Figure 10, the green area of the volcanic chart shows the metabolites with *p* < 0.05 and the absolute value of variation multiple >2 (Figure 10(a)). The box chart shows the univariate analysis and the highest ranking of metabolites among groups (the top 25 with small *p* values, Figure 10(b)). These metabolites were significantly different between the two groups. Figure 11(a) and (b) show the random forest and support vector machine (SVM) analyses, in which 15 metabolites with the highest differences were selected in each of the analyses.

#### 3.2.4 Identification of the characteristic metabolites

Ten metabolites with the same characteristic metabolic markers were statistically identified in the three analysis methods, which included taurocholic acid, citrulline, d. phenyllactic acid, l-tyrosine, benzoate, d-phenylalanine, linoleic acid, eicosadienoic acid, alpha-dimorphecolic acid, and dehydroepiandrosterone sulfate. These metabolites mainly belonged to the metabolism of fatty acids, amino acids, bile acids, and lactic acid (Table 5).

**Table 5:** Identification of different metabolites between groups

Metabolite	Metabolic pathway	Comparison of MHE and control
Citrulline	Amino acid metabolism	↑*
l-Tyrosine	Amino acid metabolism	↑*
d-Phenylalanine	Amino acid metabolism	↑*
Taurocholic	Bile acid metabolism	↑*
Benzoate	Fatty acid metabolism	↑*
Linoleic	Fatty acid metabolism	↓*
Eicosadienoic	Fatty acid metabolism	↑*
Alpha-dimorphecolic	Fatty acid metabolism	↓*
Dehydroepiandrosterone	Fatty acid metabolism	↓*
D-Phenyllactic	Lactic acid metabolism	↑*

↑: Compared with the corresponding group, the content is relatively high. ↓: compared with the corresponding group, the content is relatively low. *Statistical difference (*p* < 0.05).

#### 3.2.5 Analysis of the metabolic pathways of characteristic metabolites between the two groups

**Figure 12:**
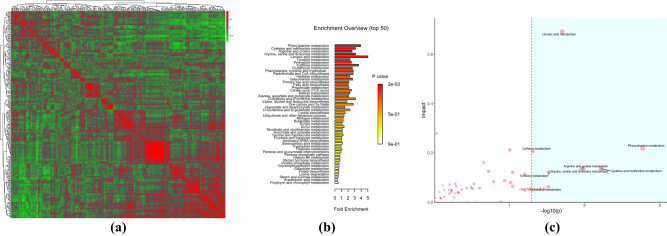
(a) Pearson correlation heat map, (b) ORA enrichment analysis, and (c) ORA topology analysis.

A comparison of the spectra of metabolite correlations of each group showed changes in metabolite correlation between the two groups. The pathological conditions might change the correlation between certain metabolites from positive to irrelevant or negative correlation, suggesting the importance of metabolite pathways. Therefore, the metabolic pathways of the intergroup characteristic metabolites were analyzed. Figure 12(a) shows that the metabolite pathways in the clustering correlation of the two groups using Pearson’s correlation analysis had a significant positive and negative correlation (red means positive correlation and green means negative correlation) between the two groups. The enrichment analysis was subsequently used to identify the biological pathways that play a key role in a biological process and reveal and understand the basic molecular mechanisms of biological processes. Figure 12(b) shows that the metabolic pathways were significantly enriched with differential metabolites (over-representation analysis (ORA) enrichment analysis), but it was still unclear whether these metabolites played a key role in the metabolic pathways. Therefore, the ORA and topological analysis (Figure 12(c)) were also performed It was observed that glycine had significant enrichment and critical roles in the metabolism of linoleic acid, phenylalanine, caffeine, arginine and proline, serine and threonine metabolism, tyrosine metabolism, and pyrimidine metabolism pathways.

#### 3.2.6 Interaction between intestinal microbiota and serum metabolites

The analysis of the intestinal microbiota and serum metabolites of the MHE patients showed that there might exist a mutual correlation; citrulline and arginine are important components of the urea cycle, and an increase in the phenylalanine and tyrosine nitration is closely associated with the ammonia levels (Table 6), conforming to the MHE “theory of plasma amino acid imbalance.” The production of intestinal ammonia is the main source of blood ammonia. In the analysis of intestinal microbiota, an increase in the abundance of *Veillonella* and a decrease in the abundance of *Lachnospiraceae, Roseburia*, and *Coprococcus* were closely correlated to the level of intestinal ammonia. Similarly, there were statistically insignificant but apparent variations in the abundance of *Faecalibacterium, Blautia, Ruminococcus*, and other genera. The abnormal metabolism of substances, such as benzoate, linoleic acid, alpha-dimorphecolic acid, and caffeine, was associated with the oxidation of fats in the body. The intestinal microbiota, including *Lachnospiraceae*, *Roseburia*, and *Coprococcus,* participated in the synthesis of butyrate, which is involved in the main pathway responsible for the breakdown of carbohydrates into SCFAs (short-chain fatty acids), inhibiting liver cholesterol and low-density lipoprotein cholesterol (ldl-c) biosynthesis. In addition, *Collinsella, Coriobacteriacea,* and *Actinomycetaceae* belonging to *Actinobacteria* were also closely related to the metabolism of polyunsaturated fatty acids, serum cholesterol, and triglycerides and participated in the metabolism of endogenous lipids. Similarly, taurocholic acid is a primary bile acid and plays an important role in the subsequent secondary and tertiary anabolism of bile acid, while the increase in its metabolism, along with the abnormal metabolism of pyrimidine, is associated with the metabolism of bile acid, as shown by an increase in the genera *Parabacteroides* and *Veillonella* in the intestines. As another example, the increase in the metabolism of serum d-phenyl lactic acid corresponds to the increase in the frequency of *Enterococcus*, *Enterobacteriaceae*, and other genera in the intestinal microbiota and that of *Ralstonia*, *Actinomyces*, *Blautia*, and *Phascolarctobacterium* in the mutual analysis of genera. All these indicated an increased level of pathogenic bacteria in inflammatory infections and that the fluctuation in bacterial genera was correlated to the butyrate metabolism *in vivo*.

**Table 6:** Interaction between intestinal flora and serum metabolites

Intestinal flora (Genus)	Metabolic pathway	Metabolites in serum
I Collinsella*	Fatty acid metabolism (I, II, III, IV, V) (①②③④)	① Benzoate*
II Faecalibacterium*		② Linoleic acid
III Blautia*		③ Alpha-dimorphecolic acid*
IV Ruminococcus*		④ Caffeine*
V Parabacterpides*	Inflammatory response (V, VI, VII) (⑤)	⑤ d-Phenylalanine*
VI Entetococcus*	Bile acid metabolism (V, VI, VII, VIII) (⑥⑦)	⑥ Pyrimidine*
VII Enterobacteriaceae*		⑦ Taurocholic acid*
VIII Veillonella*	Amino acid metabolism (VIII, IX, X, XI, XII, XIII, XIV) (⑧⑨)	⑧ Citrulline*
IX Lachnospiraceae*		⑨ Arginine*
X Roseburia**		
XI Coprococcus**		
XII Faecalibacterium*		
XIII Blautia*		
XIV Ruminococcus*		

Note: *Difference between the control group, but no statistical significance.

**Statistical significance (*p* < 0.05).

## 4 Discussion

*Veillonella* is a potentially pathogenic bacterium for MHE in general, which is one of the human oral bacteria. It can play a synergistic role after being translocated along the gastrointestinal tract into the intestine by aggravating HE [18]. It was found that the pathogenic process was mainly inhibited by the reduction in pH by the fermentation of lactic acid, which indirectly promoted the expression of pro-inflammatory cytokines in intestinal mucosa and produced endotoxin to affect intestinal immunity [19]. At the same time, the increase in ammonia production led to subtle or obvious HE, resulting in cognitive decline and an increase in the MELD (model for end-stage liver disease) score [20]. Moreover, *Veillonella* increases the production of ammonia, which is associated with endothelial activation and bile metabolism *in vivo* [21,22]. Among the top 20 strains, *Enterococcus* and *Klebsiella* were also included in the potential pathogenic genera of MHE. The increased counts of *Enterococcus* and *Klebsiella* in the intestinal mucosa of MHE patients and fecal samples of patients with cirrhosis, respectively, have been experimentally confirmed [12,23]. Meanwhile, *Enterococcus* and *Enterobacteriaceae* are pathogenic bacteria that are associated with inflammation and infection *in vivo*. It is speculated that the inflammatory response might open the blood–brain barrier (BBB), causing the toxin to enter the brain in most MHE cases [24]. *Lachnospiraceae*, *Roseburia*, and *Coprococcus*, on the other hand, play roles opposite to that of *Veillonella*. They are called beneficial bacteria and mainly participate in the production of butyrate [25]. A study has confirmed that the butyrate production by the genera *Lachnospiraceae, Roseburia*, and *Coprococcus* was mainly caused by their ability to decompose carbohydrates into SCFAs, inhibiting the synthesis of cholesterol and low-density lipoproteins in the liver [19]. Butyrate, as the main energy source of epithelial cells, can enhance the function of intestinal barriers by regulating intestinal pH, inhibiting nuclear factor-κB signaling pathway, promoting the production of mucin and antimicrobial peptides, and reducing the expression of pro-inflammatory factors and cell adhesion molecules by enhancing the integrity of intestinal epithelial cells [26,27]. Relevant evidence [21,27] shows that *Lachnospiraceae*, *Roseburia*, and *Coprococcus* are negatively correlated with the HE, the related inflammation and endothelial activation caused by the increased level of intestinal ammonia, and significantly and positively correlated with the good cognitive ability, level of intestinal immunoglobulin A, and immunity, exhibiting a good inhibitory effect on the development of HE. Similarly, the top 20 bacteria with differences in their frequencies between the MHE and control groups, including *Faecalibacterium*, *Blautia*, *Ruminococcus*, and *Ruminococcaceae*, are all correlated to the normal production of butyrate. In addition, the increase in the frequencies of families *Coriobacteriia, Coriobacteriales*, and *Coriobacteriaceae* that belong to the *Actinomyceae* was statistically significant, and the same was true for the *Actinomycetales* that belong to *Actinomyceae*. *Collinsella* (*Coriobacteriaceae*) and *Actinomycetaceae* are closely associated with the metabolism of polyunsaturated fatty acids, serum cholesterol, and triglycerides, indicating that they might be involved in the metabolism of endogenous lipids [28]. Interestingly, the subsequent analysis of species correlations found reciprocal inhibition in the genus, confirming the previous discussion. *Roseburia* and *Collinsella* are butyrate-producing bacterial genera, and *Oscillospira* was found to have significantly decreased frequency in the patients with inflammation, which might be related to the production of butyrate, and the changes in its level could lead to a decrease in the cognitive behavior and alteration in the brain neurotransmitter levels [29]. Parabacteroides have been shown to improve liver injury and regulate liver inflammation and expression of oxidative stress without causing significant steatosis [30]. They have also been found to improve glucose and lipid metabolism in mice by affecting the metabolism of intestinal bile acid and the production of succinic acid [31]. In this study, the four genera mentioned above were significantly and negatively correlated with *Rothia* in the subjects, suggesting the mutual inhibition among genera. Subsequently, the negative correlation between *Enterococcus* and *Ruminococcus* conformed to the previous discussion, with inverse effects on butyrate production. In addition, *Ralstonia* and *Actinomyces* were likely to cause infection in the intestine and promote inflammation, while *Blautia* and *Phascolarctobacterium*, which are negatively correlated with them, played an anti-inflammatory role and were significantly associated with the systemic inflammatory cytokines [32]. The subsequent prediction of community function using KEGG showed significant differences between the MHE and control groups, including differences in chromosomes, amino acids, methane metabolism-related enzymes, arginine, and proline metabolism differences (*p* < 0.05). This indicated that the occurrence and development of MHE might be correlated with the possible changes in the function of intestinal microbiota. Therefore, it was speculated that the intestinal microbiota and serology might correlate; as a result, they were analyzed using serum metabolomics.

The analysis of the serum metabolites in subjects was carried out using LC-MS/MS. After verifying significant differences in the metabolites between the MHE and control groups, 10 metabolic markers with the same differences were compared, which included taurocholic acid, citrulline, d-phenyl-lactic acid, l-tyrosine, benzoate, phenylalanine, linoleic acid, eicosapedienic acid, A-diformic acid, and dehydroepiandrosterone. As for the synthesis of blood ammonia, citrulline is mainly catalyzed by ornithine carbamoyltransferase after entering the mitochondria and transferred to the cytoplasm during the next step of arginine synthesis, known as the urea cycle, while ornithine is produced by hydrolysis [33]. In this study, as compared to the control group, a significant increase in the citrulline in the MHE group indicated an increase in blood ammonia content resulting from an increase in the ornithine cycle, consistent with the pathogenic characteristics of MHE. The increase in tyrosine metabolites in the blood in the MHE group was considered to increase acute ammonia poisoning, which induced oxidative stress in the brain, consistent with the previous results of Reinehr and Murthy. The rise in ammonia levels in the rat astrocyte culture and brain slices induced oxidation/nitrosation stress, leading to the formation of a protein tyrosine nitration (PTN) and 8-hydroxy guanosine oxidation caused by RNA, which were closely related to the patients having HE [34,35]. Tyrosine is produced by the hydroxylation of phenylalanine in the human body. Phenylalanine also increased significantly in the MHE group compared to the control group, consistent with the MHE “plasma amino acid imbalance theory.” In addition, the bile acids synthesized by cholesterol in the liver, and taurocholic acid, which increased in the MHE group, belong to primary bile acids. Taurocholic acid combines with taurine under the action of micro-mitochondrial bile acid-N-transacylase and sulfonate transferase in the cytoplasm and plays an important role in the subsequent synthesis of secondary and tertiary bile acids [36]. In chronic liver disease (cirrhosis), the synthesis of liver bile acids is reduced, and the portal vena cava bypass is opened; the bile acids are no longer confined to the enterohepatic circulation, which results in the abnormal distribution of bile acids and increased levels of bile acids in the blood. At the same time, the intestinal microbiota and metabolism of bile acids are interdependent and competitive. The abnormal metabolism of bile acids led to disturbances in the structure of intestinal microbiota, deficiency of beneficial bacteria, inflammation, and abnormal increases in ammonia levels [37,38]. Moreover, some studies have found that the abnormal bile acid signals are involved in the HE caused by acute liver function injury, including neuronal dysfunction, neuroinflammation, and BBB permeability [39]. In comparison to the control group, in the serum of the MHE group, the concentration of benzoate, linoleic acid, alpha-dimorphecolic, and acid dehydroepiandrosterone (dhea) showed that the levels of fatty acids were abnormal, which might be due to the metabolic abnormalities of bile acids (bilirubin) caused by changes in intestinal microbiota [40], or due to the damage of mitochondria caused by a decrease in the oxidation of fatty acid, posing problems in the tricarboxylic acid cycle. The d-phenyl-lactic acid, catalyzed by d-lactate dehydrogenase to produce phenylpyruvate, is a broad-spectrum antibacterial compound with antibacterial and fungal activities. The increase in the concentration of d-phenyl-lactic acid in the serum of the MHE group was considered to be correlated to the increase in the inflammation level in MHE patients. Finally, the topology analysis for the enrichment of group differences among the metabolites of metabolic pathways showed that the metabolism of linoleic acid, benzene, propane, caffeine, arginine, proline, glycine, serine, threonine, and tyrosine metabolism significantly affected the enrichment of metabolic pathways, where the analysis of the metabolites in the metabolism of linoleic acid, benzene, propane, and tyrosine was consistent with previous results. In the other four metabolic pathways, the arginine metabolism is the third step of the ornithine cycle in ammonia metabolism in MHE, where citrulline and aspartic acid are catalyzed to arginine, which then undergoes arginine hydrolysis to form urea. The metabolism of serine and threonine could promote phospholipid synthesis and fatty acid oxidation, both of which are correlated to the metabolism of fatty acids in the body [41]. Proline and glycine are commonly used as raw materials for the synthesis of essential amino acids. The metabolism of pyrimidine is correlated to the accumulation of lactic acid due to the imbalance of lactic acid metabolism in the body and the reduction of carbon and nitrogen sources caused by the abnormal metabolism of bile acids [42]. The caffeine metabolism occurs in the liver, and three different dimethylxanthines form after oxidation by the cytochrome oxidase P450 system [43]. Paraxanthine accounts for 84% of the total xanthine, which mainly accelerates lipolysis in the body and increases the content of fatty acids in the plasma, consistent with the regulation of fatty acid metabolism in the MHE group.

To sum up, the structural imbalance in the composition of intestinal microbiota in MHE patients was mainly characterized by the decrease in the beneficial bacteria at the levels of phylum, class, order, family, and genus, an increase in the pathogenic bacteria, and the imbalance in the relative frequencies of all bacteria and genera in the intestine. Special attention should be paid to the frequency changes of *Veillonella, Enterococcus, Klebsiella, Blautia, Phascolarctobacterium, Enterococcus*, and *Ruminococcus*, which might be related to the progression and deterioration of MHE. At the same time, we should pay attention to the changes in *Lachnospiraceae, Roseburia, Coprocccus, Collinsella, Blautia,* and *Phascolarctobacterium,* which are related to butyrate and lipid metabolism and have certain anti-inflammatory effects, possibly contributing to MHE improvement. The main predicted functions that showed significant differences included chromosomes, amino acid-related enzymes, methane metabolism, and arginine and proline metabolism. In LC-MS/MS study, our conclusion is to pay attention to the metabolism of fatty acids, amino acids, bile acids, and lactic acid in MHE serum, and its metabolic level might predict the prognosis of MHE. Moreover, the MHE-specific intestinal microbiota and serum metabolites were analyzed, and their mutual correlation was investigated. The interactions between bacteria due to the imbalance in the intestinal microbiota and its effects on the changes in serum metabolites, including increased inflammatory response and endotoxin level, were studied, which promoted the pathological progress of MHE. This understanding can be used for subsequent studies and can provide a basis for designing effective therapeutic strategies against MHE.
